# Mechanical and Corrosion Tests for Magnesium–Zinc/Ti-6Al-4V Composites by Gravity Casting

**DOI:** 10.3390/ma17081836

**Published:** 2024-04-16

**Authors:** Song-Jeng Huang, Chuan Li, Jun-Hang Feng, Sivakumar Selvaraju, Murugan Subramani

**Affiliations:** 1 Department of Mechanical Engineering, National Taiwan University of Science and Technology, Taipei 106336, Taiwan; hpjh101001@gmail.com (J.-H.F.); smurugan2594@gmail.com (M.S.); 2 Department of Biomedical Engineering, National Yang Ming Chiao Tung University, Taipei 112304, Taiwan

**Keywords:** gravity casting, Ti-6Al-4V alloy, uniaxial tensile test, potentiostat, electrochemical impedance spectroscopy, Hank’s balanced salt solution

## Abstract

A new Mg-4Zn X Ti-6Al-4V (TC4, of 0, 1, and 3 wt.%) alloy was successfully fabricated by a simple and low-cost gravity casting method and heat treatment at 150 °C for 24 h. The composite was examined by XRD, uniaxial tests, FESEM/EDS, potentiostat/EIS, and immersion tests for the material’s microstructures, mechanical properties, electrochemical characteristics, and corrosion resistance. Experimental results indicate that heat treatment enables the precipitation of Zn along the Mg grain boundaries and drives the co-precipitation of Al around the TC4 particles and nearby grain boundaries. Uniaxial tensile tests reveal that TC4 reinforces the Mg-Zn matrix material with higher elastic modulus, ultimate tensile stress, and toughness. The heat treatment further enhanced these mechanical properties. Electrochemical tests show that 1 wt.% TC4 composite exhibits the highest open circuit potential among all tested samples, which implies the 1 wt.% TC4-added Mg-Zn is better resistant to the oxidation of the essential metals Mg, Zn, and Al. The immersion tests in the HBSS solution further show that the 1 wt.% TC4 composite has the lowest rise of pH values after 14 days, and EDS for the corroded surface signifies that Mg is the main element vulnerable to oxidation by corrosion.

## 1. Introduction

Gravity casting is a cost-effective manufacturing process due to its simplicity in operations and flexibility in scaleup for mass production [1]. Another advantage of gravity casting is its capacity to outfit complex geometry with high precision [2,3]. For light metals such as aluminum or magnesium, gravity casting is a popular choice for making parts because of the relatively low melting points of these metals, and thus is both energy and time-efficient [4]. In this study, we choose gravity casting to prepare the Mg-Zn-based composite materials.

Mg-Zn-based composite materials have been a promising candidate for many medical implants in recent decades. For example, the bioresorbable and degradable faster Zn with the slower Mg in human bodies fit the purpose of cardiovascular stents well. Meanwhile, the mechanically weaker Zn alongside stronger Mg can meet the working requirements for vascular stents which must dilate the blocked blood vessels to increase the blood flow to achieve therapeutic effect [5,6,7,8].

In fact, magnesium-based alloys have been recognized as a next-generation, general-purpose scaffold material [6] due to their decent mechanical properties, high biocompatibility, and fast biodegradability [6,9,10].

Although Mg-Zn alloys possess good mechanical properties and biocompatibility [9], their corrosion resistance is a common problem of in vitro applications due to the high chemical activity of Mg [6,9,11]. One viable way to improve their corrosion resistance is to incorporate some metallic noble alloys of micro-size such as Ti-6Al-4V (TC4), a well-known and long-approved biomedical material [12,13,14]. These thermally stable titanium-dominant particles can strengthen the Mg-Zn matrix mechanically and relegate the available surface sites for redox reactions by alloying with different metals and thus alter the corrosion resistance. More specifically, the addition of Ti-6Al-4V powder can effectively increase the density of grain boundaries and reduce the grain size [12,13,14]. Through the formation of Mg-Zn-Al alloy compounds, it also reduces the β-phase magnesium, which is prone to be corrosive [15,16].

However, particle-reinforced Mg-Zn alloys are still at the stage of laboratory development, far from industrial products. The adding of TC4 micro-particles could involve the outset of ternary Mg-Zn-Al alloys in local areas due to the high solubility among Mg, Zn, and Al.

The ternary Mg-Zn-Al alloys have been particularly suitable for thermal analysis in metallography for many years [17,18]. This is due to the high degree of mutual solubility among aluminum, magnesium, and zinc, which eases the motion of atoms by thermal energy. It is worth mentioning that zinc has noticeably high solubility, up to 66.4%, in aluminum [19,20]. This is the essential reason for the derivation of the well-known 7000 series of zinc-containing aluminum–magnesium alloys, which has a huge number of applications in industries, such as aerospace, automobile, military, or even bioengineering utilizing the alloy’s high mechanical strength [21,22,23].

The high solubility also leads to the formation of various crystal phases in the Al-Mg-Zn alloys by heat treatments. For instance, the cubic crystal structure (T-phase, Mg_32_(Al, Zn)_49_) or the hexagonal crystal structure are due to the precipitation hardening over different levels of temperature and periods [24,25,26,27].

Another feature of Mg-Al-Zn alloys is their good corrosive resistance due to the high solubility amongst the metals to improve the homogeneity in the microstructures. The corrosion issue has been investigated intensively in the past half-century since the introduction of corrosion-resistant techniques for steel plates [28,29], which implies a new front of applications in biomedical engineering as implants or replacements.

In this study, we prepared the Mg-Zn alloy as the base material, to mix with Ti-6Al-4V particles of different weight percentages to form a composite reinforcement by gravity casting. Further T5 heat treatment was carried out to improve the mechanical strength and corrosion resistance of the cast composites [30]. The purpose of this study was to investigate the impact of TC4 microparticles on the mechanical and electrochemical behaviors of the Mg-Zn-based composites for potential bioengineering applications. All material characteristics and results are discussed in detail in the next section.

## 2. Materials and Methods

### 2.1. Raw Materials and Resistive Gravity Casting

Pure magnesium (99% purity, 7.5 kg tapped bar) and zinc (99% purity, powder) were purchased from Xintong Metals (Yangzhou, China) and First Chemical Group Co., Ltd. (Taipei, Taiwan), respectively. Titanium alloy particles (Ti-6Al-4V, TC4, *ϕ*_avg_ = 36.36 ± 23.5 μm) were purchased from Xingyjing Metal Co., Ltd. (Xingtai, China). 

Different compositions of alloy compounds in this study are listed in Table 1, where the percentage of zinc added is 4 wt.% and the addition of Ti-6Al-4V (TC4) as reinforcement is 0, 1, and 3 wt.%, with the remaining balance by the matrix Mg.

**Figure 1:**
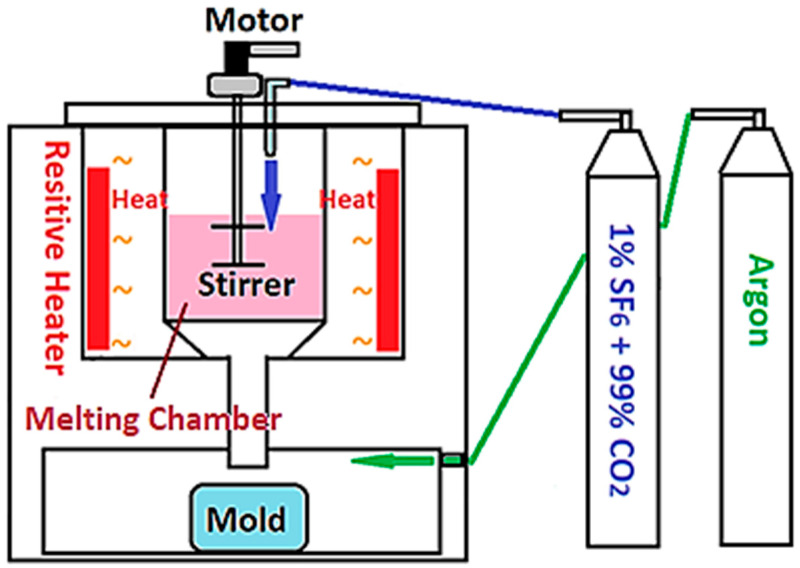
The schematic diagram of the furnace for resistive gravity casting.

All powders were cast into ingots by gravity casting. A self-made furnace for resistive gravity melting is shown in Figure 1. The casting started with heating the crucible to 400 °C, then 1% SF_6_ and 99% CO_2_ mixed gas was fed into the crucible as a protective gas to minimize the oxidation of melts. When the temperature of the crucible reached 650 °C, argon was introduced into the chamber at the bottom of the crucible to isolate the mold from the outside atmosphere. When the temperature heated up to 760 °C, the double-bladed stirrer was turned on for 10 min and then the melts were dropped into the mold by gravity. The process of gravity casting is our own system based on a set of parameters set up in the past decade. The criteria for the selection time and temperatures are subjected roughly to several conditions: (i) thorough melting; (ii) minimal oxidation; (iii) uniformity of powders; and (iv) minimum temperature between ingots and mold.

For the heat treatment after casting, we followed the standard process of heat treatment for magnesium alloys, the heating rate of the T5 (precipitation hardening) process is controlled at 20 °C/h and the temperature is maintained at 150 °C for 24 h. The selection of appropriate temperature and time is based on our own experience and the reference [30], in which the T5 was conducted for similar alloys Mg_0.94_Zn_0.06_ to achieve precipitation hardening. Note that the T5 treatment is an aging process typically at a temperature range of 120–175 °C. It aims to improve the mechanical toughness of the alloy but could lower its tensile strength and creep resistance if the heating temperature is high over a long time.

### 2.2. Surface Morphology and Chemical Compositions

Field emission scanning electron microscopy (FESEM 7900F JEOL Co., Tokyo, Japan) and energy-dispersive X-ray spectroscopy (EDS, Oxford Ultimmax 100, Oxford Instrument Co., Oxford, UK) were used to examine the surface morphology and chemical compositions of the specimen. Before examination, all specimens were cut into 10 mm × 10 mm × ~3 mm by a quartz cutter. The cut pieces were polished by 400–4000 silicon oxide sandpaper, followed by 50 nm grain size aluminum oxide particles using a grinding wheel operated at 200 rpm.

### 2.3. Microstructure

The crystal structure of the specimen was examined by an X-ray diffractometer (XRD, D2 PHASER X-ray Diffractometer, Bruker Co., Boston, MA, USA). The operation follows the standard setup by Bruker as the scanning angle 2θ: 20°–80° at 0.05°/step, the average wavelength 1.54184 Å of the anode Cu-Kα, the power 600 W, and the electric potential of ~50 kV.

Numerical fittings for diffraction peaks were implemented by DIFFRAC.EVA^®^ 5.2 (Bruker Co., Boston, MA, USA). The major database used for fitting our samples was PDF-2 2003 XRD.

### 2.4. Mechanical Testing

**Figure 2:**
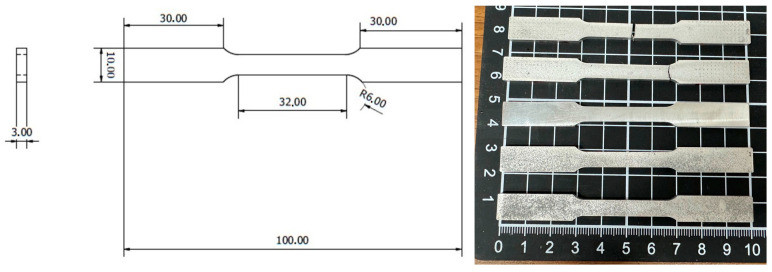
The dimension of the specimen in mm for the uniaxial tensile test following ASTM-E8-13 standard.

The specimen for uniaxial tensile testing was prepared according to ASTM-E8-13 standard as shown in Figure 2, and conducted with an MTS-810 tensile machine (MTS Co., Eden Prairie, MN, USA). The elongation speed was 1 mm/min at a precision of 0.01 kgf. Three samples were prepared for the tensile tests in each case (0, 1, and 3 wt.% TC4, as-cast and heat-treated).

The strain was measured by the built-in hydraulic system (Hydraulic Collet Grips MTS-646) based on the displacement of specimens. The testing procedure also follows the ASTM-E8-13 standard.

The hardness of materials was measured by the Vickers microhardness tester using Wilson’s VH1102/1202 (Buehler, Lake Bluff, IL, USA). The pyramidal diamond indenter of 136° was used under the loading set at 0.1 kgf/mm^2^ for all tests.

### 2.5. Electrochemical Tests

The electrochemical tests (potentiodynamic polarization (potentiostat) and electrochemical impedance spectroscopy (EIS)) for sample materials were conducted on an AUTOLAB PGSTAT128N (Metrohm AG, Herisau, Switzerland) in Hank’s balanced salt solution (HBSS) with pH values of 7.4 ± 0.2, at a temperature of 37 °C. The three electrodes were, respectively, a magnesium alloy sample as the working electrode, Ag-AgCl as the counter electrode, and platinum as the reference electrode. Note that only 1/20 of the sample area was exposed to HBSS solution throughout the tests. This was meant to keep the integrity of the working electrode. The chemical compositions of the HBSS solution composition are listed in Table 2 following the formulation from [31]. 

For the test of potentiostat, the range of voltage scan was between around −1.6 V to −1.4 V across the open circuit potential (OCP) at a scan rate of 0.0010681 V/s. This range was chosen based on rounds of trials and the reference silver chloride electrode (E = +0.197 V in saturated KCl).

EIS was measured at OCP from the results of potentiodynamic polarization, covering a frequency range from 100,000 Hz to 0.1 Hz. The EIS data were collected using the software NOVA^®^ 2.1.6 (Metrohm, Co., Herisau, Switzerland) at a rate of 10 points per decade change in frequency. The numerical fitting for EIS by an equivalent circuit was conducted by ZSimpWin^®^ (AMETEK Inc., Berwyn, PA, USA).

### 2.6. Immersion Test

The HBSS solution (pH value of 7.4 ± 0.2, Table 2) was used as the immersion medium for sample materials. We kept 1/20 of the sample area exposed to HBSS solution throughout the tests. A constant ambient temperature of 37 °C was maintained in a customized oven. Variations of pH values were measured every 24 h and the immersion lasted for 14 days. The pH meter used for measurement was a pH 510 (Eutech Instruments Pte. Ltd., Singapore).

## 3. Results

### 3.1. Surface Morphology

**Figure 3:**
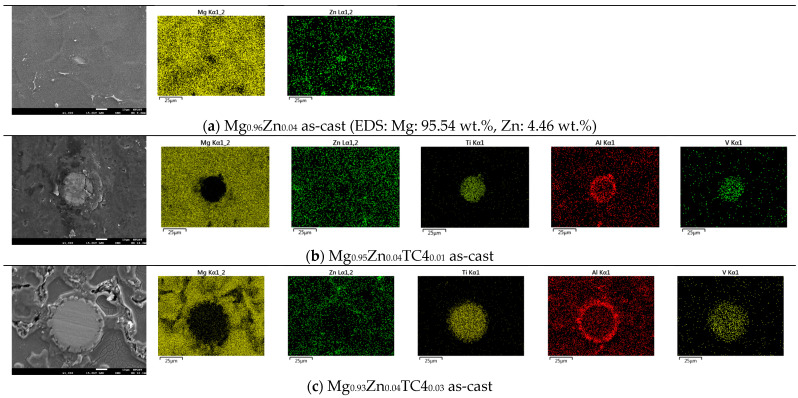

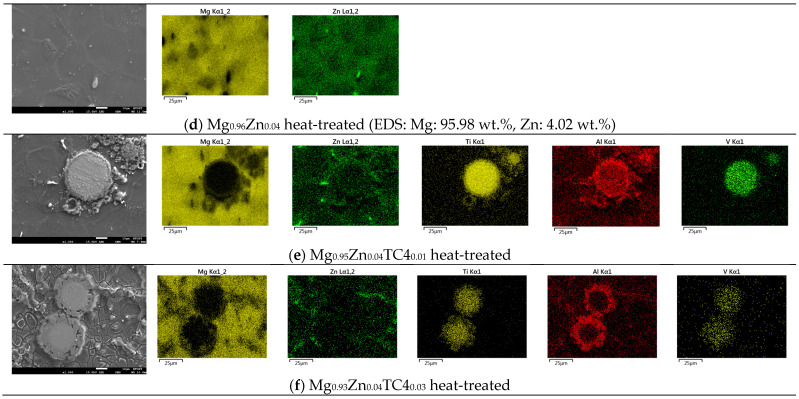
FESEM images and EDS mapping (1000×) of the samples. The heat treatment is set at 150 °C for 24 h. For the zero-TC4 (non-reinforced) samples, the elemental weight percentages of Mg and Zn are presented as a cross-reference to the original input powders in Table 1.

Figure 3 shows the FESEM and EDS of sample materials. We particularly present the images of embedded TC4 particles to illustrate the distribution change of chemical elements by the heat treatment. All FESEM images have a magnification of 1000X along with elemental analysis by EDS.

One important feature observed in these images is that the precipitation of Zn can be found along the grain boundaries. In all presented cases, the precipitation of Zn out of the Mg matrix is visible, especially prominent in the heat-treated samples and accordingly, these images corroborate the out-diffusion of Zn from the Mg matrix.

Nonetheless, EDS also reveals that the heat treatment affects the diffusion of Al. For samples of 1 wt.% and 3 wt.% TC4, Al can be found around the edge of TC4 particles, whereas Ti and V stand still within the particles. It too happens that Zn tends to surround the TC4 particles in those cases. Since the zinc–aluminum alloy has been a well-known alloy for a long time [17,18], we speculate that the diffusion of zinc can also displace aluminum due to its mutual solubilities. The less dense and more separated TC4 particles at 1 wt.% in the Mg matrix allow easier diffusion of Al and Zn, whereas a denser distribution of TC4 particles at 3 wt.% would reduce the mobility of Zn and Al. Consequently, Zn is more restricted to moving a shorter distance within grains.

### 3.2. Crystal Structure

**Figure 4:**
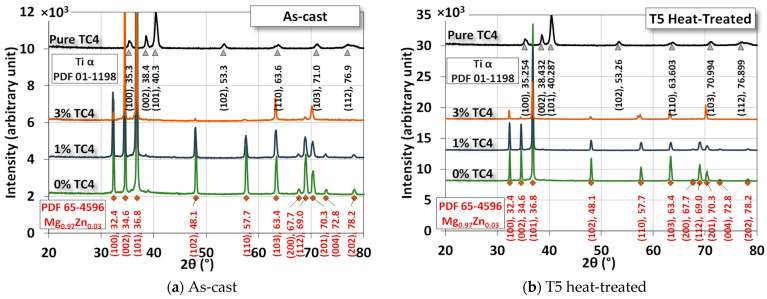
XRD of the sample materials. The heat treatment is set at 150 °C for 24 h.

Figure 4 shows the XRD of the sample materials before and after heat treatment. The difference due to heat treatment is very minor as the treatment is only set at 150 °C, far below any phase change points among these metals. The numerical fitting by Gaussian functions can be identified by PDF# 65-4596 from the database for the crystal structure of Mg_0.97_Zn_0.03_, which is very close to our original compositions of powders in Table 1. The most identified peaks for pure TC4 belong to Ti-α (hexagonal close-packed, HCP). However, since the weight percentage of TC4 is low, Ti peaks are invisible in the MgZn-TC4 composite. 

The most prominent peaks in the composite are (100), (002), (101), (102), (110), and (103). The intensities of these peaks were reduced by the increased amount of TC4, but their presence is certain by numerical fitting. 

XRD assures us that the crystal structure remains almost intact by the heat treatment, although we observe some precipitations of Zn and Al in the FESEM images. This implies that the main crystal structures are stable, and precipitation only happens locally due to the heat treatment.

### 3.3. Uniaxial Tensile Test

**Figure 5:**
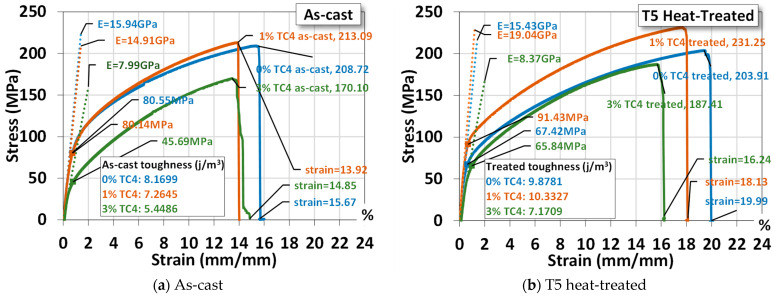
Uniaxial tensile tests for a sample material. The symbol E stands for the elastic modulus and the yield stress is estimated by 0.2%-offset based on the elastic modulus. The heat treatment is set at 150 °C for 24 h.

An example of the uniaxial tensile tests of sample materials is shown Figure 5, where materials that underwent heat treatment exhibit higher ultimate stresses, larger fracture strains, and higher Young’s modulus and toughness (by numerical area integration). The one-day heat treatment (24 h), though only at 150 °C, delivers a hardening effect on the mechanical properties. It is interesting to notice that both Mg-Zn alloys and Mg-Zn-TC4 composites become more ductile (larger elongation) and tougher (higher strain energy) simultaneously. This change can be useful for practical uses, as the ductility and toughness usually vary oppositely by the process of heat treatment. We think that the low-temperature, 24 h-long treatment can be beneficial in causing minor changes in microstructures at a much slower pace.

For a comparison, the added 1 wt.% TC4 does reinforce the Mg-Zn alloys mechanically. But samples of 3 wt.% TC4 have mechanical properties inferior to the Mg-Zn alloys and 1 wt.% TC4 composites as well. This could be related to the different patterns of precipitation observed in the FESEM, where the co-precipitations of Zn and Al for TC4 3 wt.% become less prominent and thus weaken the reinforcement and demote the mechanical strength as well.

The statistical data of three samples for each case (0, 1, and 3 wt.% TC4, as-cast and heat-treated) are listed in Table 3 for readers’ reference. We shall use these average values for comparisons with other studies later. 

### 3.4. Microhardness Test

**Figure 6:**
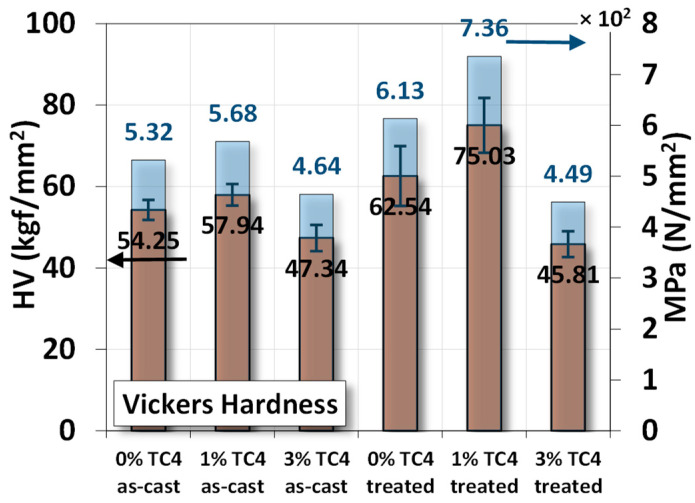
Vickers hardness measurements for sample materials. The heat treatment is set at 150 °C for 24 h.

The Vickers hardness measurements of sample materials are shown the Figure 6. The heat-treated samples have higher hardness compared to their respective as-cast ones, and the added TC4 reinforces the hardness of Mg-Zn matrices.

Using the measured hardness in N/mm^2^ (MPa), we estimated ultimate tensile strength (*σ_u_*) of the material can be approximated by the following empirical formula [32,33,34]:

HV (MPa)/4 ≲ *σ_u_* ≲ HV (MPa)/2 (1)

The numerical values of denominators 2 and 4 are estimated from a function of yield strength, Poisson’s ratio, work-hardening exponent, and geometrical factors in general. Based on this empirical formula, we list numerical values of ultimate strength from the uniaxial tensile test and Equation (1) in Table 4 for comparison. We find that the ultimate strength from the uniaxial tensile tests is close to the *averages* of the two bonds estimated from Vickers hardness in Figure 6 using Equation (1).

### 3.5. Electrochemical Test

**Figure 7:**
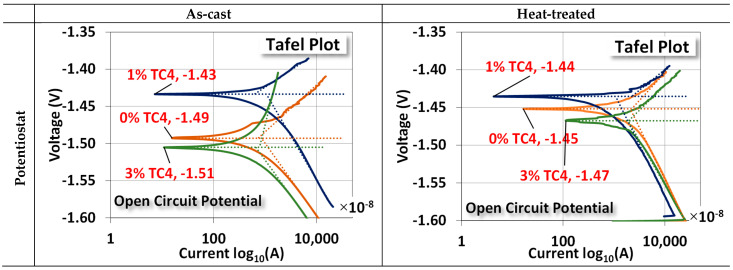

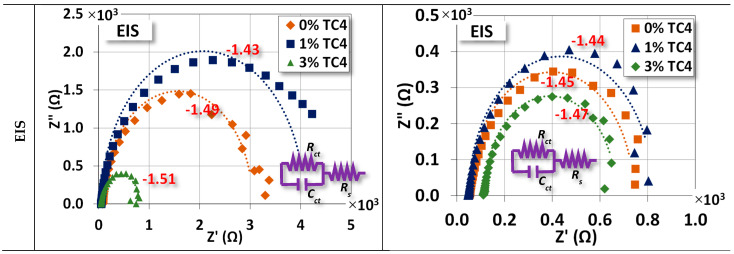
Potentiostat and EIS measurements for sample materials. The EIS was measured at the OCP for respective compositions and the heat treatment was set at 150 °C for 24 h. An equivalent circuit (Randles model) for the EIS fitting is also presented.

The two electrochemical tests, namely, the potentiostat for the open circuit potential (OCP) and the EIS, are shown in Figure 7. For OCP, the changes between as-cast and treated samples are (0 wt.% TC4: −1.49 → −1.45 V), (1 wt.% TC4: −1.43 → −1.44 V), and (3 wt.% TC4: −1.51 → −1.47 V). The case of 1 wt.% TC4 has changed slightly towards more negative voltages as compared to the other two cases. The shift of OCP toward a more negative voltage means the tested material becomes more anodic (prone to oxidized).

Overall, the heat treatment makes samples more cathodic (prone to reduction). This could be attributed to the possible formation of some Zn-Al alloys around TC4, which can (i) protect the interfaces between TC4 particles and Mg-Zn matrix from cleavages by oxidation; (ii) make the Mg less available by oxidation on the surface [35]. The much more positive reduction potential of Zn-Al alloys than that of Mg makes the composite less vulnerable to oxidation. A short discussion about the electrochemical reduction potential shall be presented later.

EIS shows that the sample materials after heat treatment become electrically less resistant and reactant (lower Z′ and Z″, note the scale in abscissa) at their respective OCP. This reduction implies that samples could have a faster response to the chemical reactions on the surface in terms of frequency. 

The numerical values of each component in the equivalent circuit (Randles model) are listed in Table 5 where the *R_s_*, *R_ct_*, and *C_ct_* roughly represent the resistance of the diffuse layer in the electrolyte and the resistance and capacitance of the electrical double layer (Helmholtz) near the surface, respectively. Among all samples, we notice that *R_ct_* and *C_ct_* for 1 wt.% TC4 samples are highest among the as-cast and the heat-treated groups, respectively. This implies that the charges transferred with the electrical double layer near the surface of 1 wt.% TC4 samples are more difficult than other samples. From the prospect of redox reactions, it indicates more challenging for redox reactions to occur because redox reactions are primarily the transfer of electrons among charged particles.

### 3.6. Immersion Test

**Figure 8:**
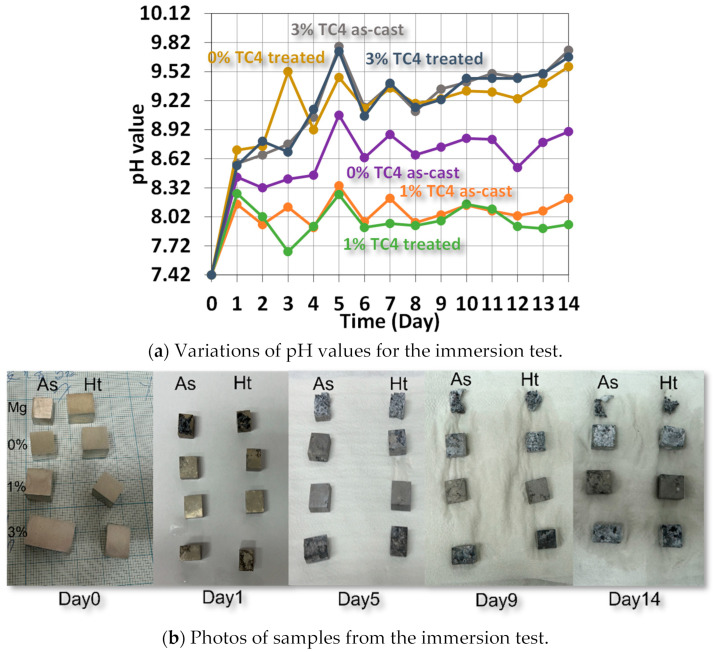
Variations of pH values from the immersion test in HBBS for sample materials. The sample materials after immersion are shown in the photos. The heat treatment is set at 150 °C for 24 h.

Figure 8 shows the variations of pH values for samples immersed in HBSS solution for up to 14 days, when we can find the elevated pH values due to the release of metallic ions such as Mg, Zn, or even Al via complex reactions between the compositions of HBSS and sample materials. The higher pH values imply more release of metallic ions via the surface chemical reactions with the HBSS. In other words, The higher the pH, the higher the likelihood of corrosion on the surface. Qualitatively, we can order the extent of corrosion following the pH values at the end of the test as 1% TC4 as-cast < 1% TC4 treated < 0% TC4 as-cast < 0% TC4 treated < 3% TC4 treated < 3% TC4 as-cast. This order is close to the OCP measured in the potentiostat tests, except for the swap between 0% TC4 as-cast and 0% TC4 treated. The samples of 0 wt.% TC4 result in a higher pH value than that of samples with 1 wt.% TC4 during immersion could be caused by more extensive oxidation of Mg. Note that TC4 microparticles provide Zn, Al, and transition metals Ti, and V into the Mg-Zn matrix; these metals not only have higher standard reduction potential (less likely to be oxidized) but also reduce the surface area of Mg exposed to the corrosive sources.

## 4. Discussion

### 4.1. Precipitation of Zn and Al by Heat Treatment 

**Figure 9:**
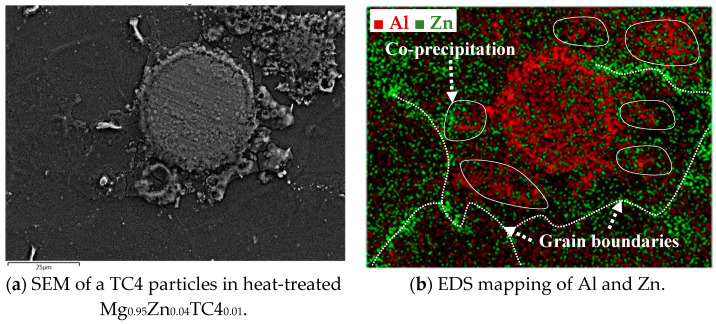
The overlaid Zn and Al in EDS images for heat-treated Mg_0.95_Zn_0.04_TC4_0.01_. The heat treatment is set at 150 °C for 24 h.

The precipitation of Zn and Al by the heat treatment is further detailed herein. Using the case of heat-treated Mg_0.95_Zn_0.04_TC4_0.01_, we overlay the EDS images of Zn and Al as shown in Figure 9, in which it is visible that the precipitation of Zn along the grain boundaries of Mg matrix and Al encircles the TC4 particle. A remarkable phenomenon is the association of Al to Zn found near the grain boundaries where Zn precipitated. This can be the zinc–aluminum alloys as mentioned previously. It is interesting to know that the alloying of Zn-Al can be found even on a small scale.

### 4.2. Multiscale Cross-Sectional Fractography of Heat-Treated Samples

**Figure 10:**
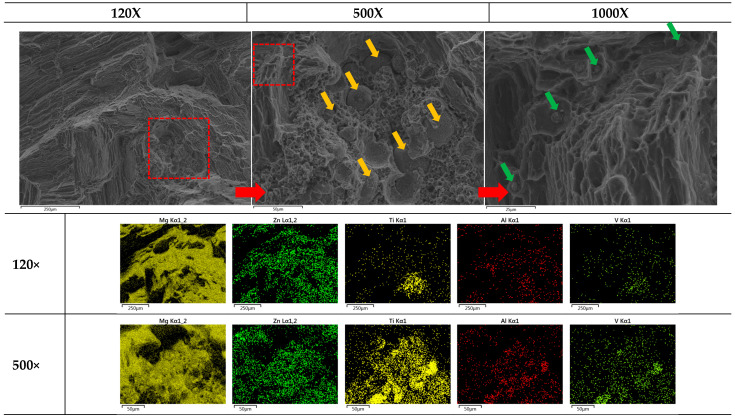

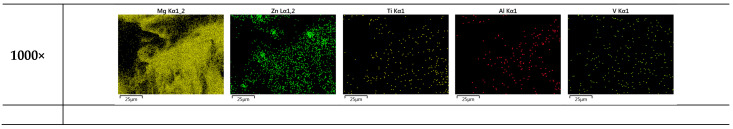
Multiscale EDS images by FESEM for heat-treated Mg_0.95_Zn_0.04_TC4_0.03_. The red square marks the zoom-in region for the magnification. Yellow and green arrows, respectively, mark the locations of TC4 and Zn particles. The heat treatment is set at 150 °C for 24 h.

The failure of sample materials is of great interest for realistic applications to understand the limits of composites. Figure 10 shows multiscale magnifications (120×, 500×, and 1000×) of heat-treated Mg_0.95_Zn_0.04_TC4_0.03_. This set of FESEM provides us with a good visualization for the embedded TC4 particles (*ϕ*_avg_ = 36.36 ± 23.5 μm) and Zn particle (~5 μm) within the Mg matrix. 

Based upon these images and previously observed co-precipitation of Zn and Al, the composite fabricated in this study can be schematically illustrated in Figure 10, where two different sizes of particles, namely the larger TC4 and smaller Zn, are distributed throughout the materials. With the assistance of Zn-Al encircling around TC4, and Zn along the grain boundaries, the Mg matrix is securely refinanced but still retains its flexibility.

### 4.3. Electrochemical Potential for the Corrosion

Identifying the element that is most likely to be oxidized among the three active metals, Mg, Al, and Zn, could provide information on the protective mechanism during the electrochemical test and even in the in situ corrosion. The standard reduction potentials for the half-reaction of AgCl (reference electrode), Zn, Al, and Mg against the standard hydrogen electrode (SHE, *E*^0^ = 0.0 V) are [30,31,36,37,38,39,40]:

(2) $$AgCl+e^{-}\to Ag+{Cl}^{-}\left(E^{0}=+0.2223\;V,\;\mathrm{reference}\;\mathrm{electrode}\right)$$

(3) $${Zn}^{2+}+2e^{-}\to Zn\left(E^{0}=-0.7618\;V\right)$$

(4) $${Al}^{3+}+3e^{-}\to Al\left(E^{0}=-1.662\;V\right)$$

(5) $${Mg}^{2+}+2e^{-}\to Mg\left(E^{0}=-2.372\;V\right)$$

where we notice that Mg has the *lowest* potential and is therefore easier to oxidize (lost electrons) compared to Zn and Al. Therefore, it seems that the most vulnerable element in the composite subject to corrosion should be Mg. This very preliminary analysis indicates that the corrosion of samples in HBSS could start with the oxidation of Mg. However, if water is taken into account, then the following reaction could occur:

(6) $$H_{2}O+e^{-}\to \frac{1}{2}H_{2}+{OH}^{-}\left(\mathrm{cathode}\;\mathrm{reaction},\;E^{0}=-0.8277\;V\right)$$

Combining Equation (5) as an anodic half-reaction and Equation (6) as a cathodic half-reaction, we have

(7) $$Mg+2H_{2}O\to H_{2}+Mg({OH)}_{2}\left(\mathrm{overall}\;\mathrm{reaction},\;E^{0}=+0.7166\;V\right)$$

This implies that a major compound in the corrosion test could be magnesium hydroxide (Mg(OH)_2_). Another possible reaction following the formation of Mg(OH)_2_ can be

(8) $$Mg({OH)}_{2}+2e^{-}\to Mg+2{OH}^{-}\left(\mathrm{overall}\;\mathrm{reaction},\;E^{0}=-2.687V\;\right)$$

But this reaction is much less likely because it has a relatively lower reduction potential and needs a high temperature to achieve.

**Figure 11:**
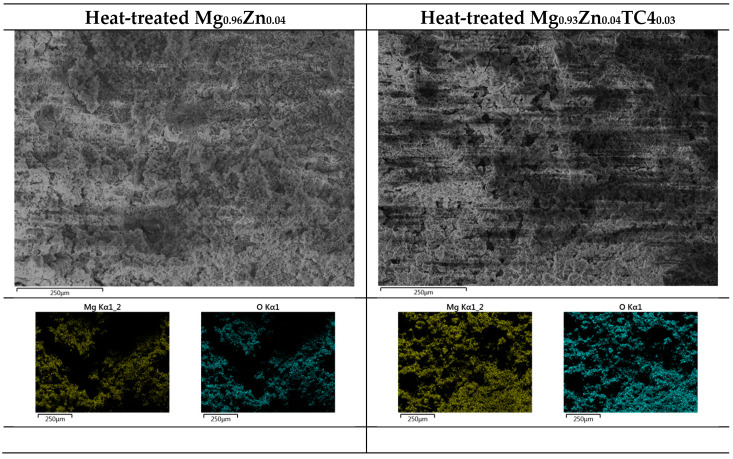
EDS images by FESEM for heat-treated Mg_0.96_Zn_0.04_ and Mg_0.93_Zn_0.04_TC4_0.03_ after 24 h immersion in HBSS at 37 °C. The surface is dominated by Mg and O.

Figure 11 shows the FESEM images of rotten surfaces for samples of 0 wt.% and 3 wt.% TC4, where compounds of magnesium oxide heavily covered the original raw materials. The EDS provides further evidence of oxidized Mg by the 24 h immersion in the HBSS solution.

Lastly, we compare the electrochemical measurements of some similar Mg-Zn-based alloys with our results in Table 6. It certainly shows that the open circuit potentials of our samples are close to other similar Mg-Zn-based alloys even though the solutions are different. This may be due to the redox reaction on the surface being almost dominated by Mg. Nevertheless, we should point out that, in any case, this comparison is for reference only since distinct manufacturing approaches and solutions can fundamentally influence the sample surface conditions and thus the open circuit voltage and corrosion current. 

### 4.4. The Correlation between Vickers Hardness and Ultimate Tensile Strength

**Figure 12:**
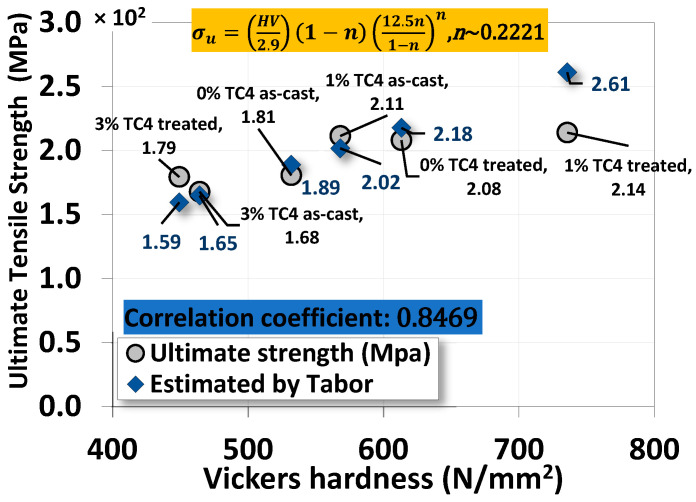
The correlation between the ultimate strength from the uniaxial tensile test and Vickers microhardness by Tabor’s empirical equation.

A simple and effective correlation between Vickers hardness and ultimate tensile strength can be Tabor’s empirical equation [32,33,34]

(9) $$\sigma_{u}=(H/2.9)(1-n){\left(\frac{12.5n}{1-n}\right)}^{n}$$

where *σ_u_* is the ultimate strength in MPa, *H* is the Vickers hardness in MPa, and *n* is the strain hardening coefficient, usually less than 1. Skipping details and derivations, we can find that the correlation between our uniaxial tensile test data and Vickers hardness is close to linear (correlation coefficient ~0.85 with the optimal strain hardening coefficient *n*~0.22 determined numerically), as shown in Figure 12. This result is quite impressive for such a simple empirical formula proposed by Tabor over a half-century ago.

### 4.5. Some Comparisons of Mechanical Properties of Magnesium–Zinc Alloys

In this section, we selected some closely similar Mg_1−x_Zn_x_-based alloys in the literature to compare their mechanical properties [42,43,44,45,46,47,48,49,50,51]. As listed in Table 7, the composite materials in our current study have slightly higher UTS (MPa) and larger elongation (%) among selected cases in the literature. It suggests that the Mg-Zn matrix can be strengthened by the added TC4 particles with enhanced ductility.

## 5. Conclusions

The magnesium–zinc/Ti-6Al-4V (TC4, 0, 1, and 3 wt.%) composites fabricated by gravity casting were examined for the materials’ microstructures, mechanical properties, electrochemical characteristics, and corrosion resistance. Experimental results indicate several features of the composites:

- XRD checks the presence of Mg_0.97_Z_0.03_ as the main crystal structures in sample materials.
- FESEM and EDS images indicate that Zn precipitated along the Mg grain boundaries for sample materials that underwent heat treatment at 150 °C for 24 h.
- Uniaxial tensile tests demonstrate that TC4 reinforces the Mg-Zn matrix material with higher elastic modulus, ultimate tensile stress, and toughness. The heat treatment further enhanced these mechanical properties.
- The heat treatment also drives the co-precipitation of Zn and Al proximally to the Mg grain boundaries.
- In the potentiostat test, the 1 wt.% TC4 composite exhibits the highest open circuit potential among all tested samples, which implies the 1 wt.% TC4 added Mg-Zn is better resistant to the oxidation of the essential metals Mg, Zn, and Al.
- EIS shows that heat-treated samples have a lower impedance of Z′ and Z″. The 1 wt.% samples have higher charge transfer resistance in the electrical double layer from the numerically fitting using the equivalent circuit (Randles model).
- For the immersion tests in HBSS solution, the 1 wt.% TC4 composite has the lowest rise of pH values after 14 days. Preliminary analysis from basic electrochemical potentials and EDS of the corroded surface signifies that Mg is the main element vulnerable to oxidation by corrosion.

## Figures and Tables

**Table 1:** Compositions of compounds under study.

Alloys	Mg (wt.%)	Zn (wt.%)	Ti-6Al-4V (wt.%)
Mg_0.96_Zn_0.04_ (0 wt.% TC4)	96	4	0
Mg_0.95_Zn_0.04_TC4_0.01_ (1 wt.% TC4)	95	4	1
Mg_0.93_Zn_0.04_TC4_0.03_ (3 wt.% TC4)	93	4	3

**Table 2:** Chemical compositions of Hank’s balanced salt solution.

Chemicals	Concentration (g/L)
NaCl	8.00
KCl	0.40
CaCl_2_	0.14
MgSO_4_ 7H_2_O	0.06
MgCl_2_ 6H_2_O	0.10
Na_2_HPO_4_ 12H_2_O	0.06
KH_2_PO_4_	0.06
C_6_H_12_O_6_	1.00
NaHCO_3_	0.35

**Table 3:** Statistics (sample mean ± standard deviation) of the uniaxial tensile tests for different sample materials. The yield strength is estimated by 0.2% offset of the elastic strain.

Samples	Yield Strength (MPa)	Ultimate Tensile Strength (MPa)	Elongation (%)	Young’s Modulus (GPa)
Mg_0.96_Zn_0.04_ as-cast	73.93 ± 6.09	180.64 ± 38.87	15.11 ± 6.58	15.68 ± 0.21
Mg_0.95_Zn_0.04_ TC4_0.01_ as-cast	79.28 ± 4.02	211.39 ± 4.86	15.31 ± 2.18	17.07 ± 1.63
Mg_0.93_Zn_0.04_ TC4_0.03_ as-cast	53.39 ± 9.15	167.74 ± 2.30	14.36 ± 1.65	6.46 ± 1.32
Mg_0.96_Zn_0.04_ treated	71.40 ± 21.99	208.01 ± 13.27	14.72 ± 2.72	14.94 ± 0.43
Mg_0.95_Zn_0.04_ TC4_0.01_ treated	91.48 ± 0.84	214.00 ± 31.76	16.14 ± 6.54	17.92 ± 1.82
Mg_0.93_Zn_0.04_ TC4_0.03_ treated	69.55 ± 13.19	144.76 ± 17.51	12.95 ± 2.43	12.16 ± 1.97

**Table 4:** Comparison of the ultimate strength between the uniaxial tests and empirical formula in Equation (1). The upper and lower bonds are calculated from the results of Vickers hardness in Figure 6.

	Ultimate Strength (MPa)		
Sample Materials	Uniaxial Test	HV Lower Bond	HV Upper Bond
Mg_0.96_Zn_0.04_ as-cast	180.64	133.00	266.01
Mg_0.95_Zn_0.04_ TC4_0.01_ as-cast	211.39	142.05	284.10
Mg_0.93_Zn_0.04_ TC4_0.03_ as-cast	167.74	116.06	232.12
Mg_0.96_Zn_0.04_ treated	208.01	153.33	306.65
Mg_0.95_Zn_0.04_ TC4_0.01_ treated	214.00	183.95	367.90
Mg_0.93_Zn_0.04_ TC4_0.03_ treated	179.29	112.31	224.62

**Table 5:** Numerical values of the equivalent circuit in EIS.

Sample Materials	*R_s_* (Ω)	*R_ct_* (Ω)	*C_ct_* (F)	χ^2^ Errors
Mg_0.96_Zn_0.04_ as-cast	83.49	3.13 × 10^−5^	2965	0.005777
Mg_0.95_Zn_0.04_ TC4_0.01_ as-cast	54.12	5.815 × 10^−5^	4021	0.1009
Mg_0.93_Zn_0.04_ TC4_0.03_ as-cast	53.59	2.321 × 10^−5^	760.5	0.005583
Mg_0.96_Zn_0.04_ treated	55.53	3.183 × 10^−5^	686	0.007858
Mg_0.95_Zn_0.04_ TC4_0.01_ treated	50.03	3.603 × 10^−5^	774.2	0.006008
Mg_0.93_Zn_0.04_ TC4_0.03_ treated	114.4	3.27 × 10^−5^	553.9	0.001945

**Table 6:** Comparison among electrochemical measurements of similar Mg_1−x_Zn_x_ based alloys from the literature.

Alloy	Fabrication Method	Open Circuit Potential (V)	Corrosion Current (A)	Solution	Source
Mg_0.96_Zn_0.04_	As-cast	−1.49	1.51764 × 10^−6^	HBSS	This work
Mg_0.95_Zn_0.04_TC4_0.01_	As-cast	−1.43	5.32837 × 10^−7^	HBSS	This work
Mg_0.93_Zn_0.04_TC4_0.03_	As-cast	−1.51	2.17 × 10^−6^	HBSS	This work
Mg_0.96_Zn_0.04_	T5 heat treatment	−1.45	9.76868 × 10^−7^	HBSS	This work
Mg_0.95_Zn_0.04_TC4_0.01_	T5 heat treatment	−1.44	1.79291 × 10^−6^	HBSS	This work
Mg_0.93_Zn_0.04_TC4_0.03_	T5 heat treatment	−1.47	4.75769 × 10^−6^	HBSS	This work
Mg_0.94_Zn_0.06_	Hot extrusion	−1.56	1.5 × 10^−7^	Normal saline	[41]
Mg_0.948_Zn_0.0395_Ca_0.005_Mn_0.0075_	Homogenization	−1.66	6.59 × 10^−6^	SBF	[42]
Mg_0.948_Zn_0.0395_Ca_0.005_Mn_0.0075_	Hot extrusion	−1.55	4.36 × 10^−6^	SBF	[42]
Mg_0.948_Zn_0.0395_Ca_0.005_Mn_0.0075_	One-pass HECAP	−1.52	3.41 × 10^−6^	SBF	[42]
Mg_0.948_Zn_0.0395_Ca_0.005_Mn_0.0075_	Two-pass HECAP	−1.49	2.57 × 10^−6^	SBF	[42]
Mg_0.947_Zn_0.04_Ca_0.005_Mn_0.008_	As-cast	−1.488 ± 0.2	4.105 × 10^−6^ ± 0.26	Hank’s solution	[43]
Mg_0.947_Zn_0.04_Ca_0.005_Mn_0.008_	Homogenization (24 h)	−1.489 ± 0.004	3.939 × 10^−6^ ± 0.25	Hank’s solution	[43]
Mg_0.96_Zn_0.04_	Hot extrusion	−1.464	3.97 × 10^−5^	PBS	[44]
Mg_0.955_Zn_0.04_Mn_0.005_	Hot extrusion	−1.381	8.1 × 10^−6^	PBS	[44]
Mg_0.95_Zn_0.04_Mn_0.01_	Hot extrusion	−1.387	9.3 × 10^−6^	PBS	[44]

**Table 7:** Comparison among mechanical properties of similar Mg_1−x_Zn_x_-based alloys from the literature.

Alloy	Fabrication Method	Yield Strength (GPa)	UTS (MPa)	Elongation (%)	Source
Mg_0.95_Zn_0.04_TC4_0.01_	As-cast	79.28 ± 4.02	211.39 ± 4.86	15.31 ± 2.18	This work
Mg_0.93_Zn_0.04_TC4_0.03_	As-cast	53.39 ± 9.15	167.74 ± 2.30	14.36 ± 1.65	This work
Mg_0.95_Zn_0.04_TC4_0.01_	T5 heat treatment	91.48 ± 0.84	214.00 ± 31.76	16.14 ± 6.54	This work
Mg_0.93_Zn_0.04_TC4_0.03_	T5 heat treatment	69.55 ± 13.19	144.76±17.51	12.95±2.43	This work
Mg_0.94_Zn_0.06_	Hot extrusion	169.5 ± 3.6	279.5 ± 2.3	18.8 ± 0.8	[45]
Mg_0.92_Zn_0.04_Gd_0.04_	As-cast	102.5 ± 5	169.4 ± 8	7.23 ± 0.3	[42]
Mg_0.92_Zn_0.04_Gd_0.04_	Solution treatment	96.9 ± 5	170.3 ± 8	8.840.3	[42]
Mg_0.948_Zn_0.0395_Ca_0.005_Mn_0.0075_	Homogenization	60	140	11.51	[43]
Mg_0.948_Zn_0.0395_Ca_0.005_Mn_0.0075_	Hot extrusion	135	238	14.53	[43]
Mg_0.948_Zn_0.0395_Ca_0.005_Mn_0.0075_	One-pass HECAP	145	246	15.82	[43]
Mg_0.947_Zn_0.04_Ca_0.005_Mn_0.008_	As-cast	170	224	1.8	[46]
Mg_0.947_Zn_0.04_Ca_0.005_Mn_0.008_	Homogenization	148	210	6	[46]
Mg_0.95_Zn_0.04_Ca_0.005_RE_0.005_	Homogenization	92	167	6	[47]
Mg_0.96_Zn_0.04_	As-cast	43.0	153.1	13.4	[48]
Mg_0.96_Zn_0.04_	Solution treatment	40	163	15	[49]
Mg_0.956_Zn_0.04_Ca_0.004_	Four-pass HECAP	99	240	17	[49]
Mg_0.954_Zn_0.04_Ca_0.006_	Hot extrusion	158 ± 3.6	260 ± 2.4	9.7 ± 1.4	[49]
Mg_0.96_Zn_0.04_	Hot extrusion	198.4	290	33.9	[50]
Mg_0.9437_Zn_0.045_Ca_0.013_	Hot extrusion	173 ± 6	251 ± 6	22.7 ± 3.0	[51]

## Data Availability

The raw data supporting the conclusions of this article will be made available by the authors upon request.
